# Robotic versus laparoscopic pancreatoduodenectomy across the learning curve: a systematic review and meta-analysis

**DOI:** 10.1007/s00423-026-04108-0

**Published:** 2026-06-19

**Authors:** Ahmed Abdelsamad, Khaled Mohamed, Youssef Badie, Zainab Hussein, Mennatullah Mohsen, Osama Selim, Omar Mohamed, Mohammed Khaled Mohammed, Zeyad M. Wesh, Felix Nickel, Sascha Vaghiri, Florian Gebauer

**Affiliations:** 1 Department of Surgery II, University of Witten/Herdecke, 58455 Witten, Germany; 2 Deputy head of the oncological surgery department, Section head of robotic surgery, Knappschaft Vest-Hospital, 45657 Recklinghausen, Germany; 3 General surgery resident at Cairo University Hospitals, Cairo University, Cairo, Egypt; 4 Faculty of Medicine, Alexandria University, Alexandria, Egypt; 5 Faculty of Medicine, Minia University, Minia, Egypt; 6 Medical intern at faculty of Medicine, Al-Azhar University, Cairo for girls, Egypt; 7 Faculty of Medicine, Cairo University, Giza, Egypt; 8 Medical intern at faculty of Medicine, Ain Shams University, Cairo, Egypt; 9 Department of Surgery, University of Hamburg-Eppendorf, Hamburg, Germany; 10 Department of Surgery (A), Heinrich-Heine-University, Medical Faculty and University, Hospital Duesseldorf, Moorenstr. 5, Duesseldorf, 40225 Germany; 11 Department of Surgery, Helios University Hospital Wuppertal, Wuppertal, Germany

**Keywords:** Robotic pancreatoduodenectomy, Laparoscopic pancreatoduodenectomy, Whipple operation, Learning curve

## Abstract

**Background:**

Minimally invasive pancreatoduodenectomy (MIPD) has a substantial learning curve. This systematic review and meta-analysis evaluated learning-phase effects in robotic (RPD) and laparoscopic pancreatoduodenectomy (LPD).

**Methods:**

The protocol was registered in PROSPERO (CRD420261283406). PubMed, Scopus, and Web of Science were searched through June 1, 2026. Studies reporting early-versus-late learning-phase outcomes or phase-specific RPD-versus-LPD comparisons were included. Random-effects models were used, and the certainty of evidence was assessed using GRADE.

**Results:**

Twenty-five studies were included qualitatively and 20 quantitatively, comprising 4,764 patients. The median case volume required to overcome the learning curve was similar for RPD and LPD (31.5 vs. 35 cases; *p* = 0.56). Late-phase MIPD was associated with significantly shorter operative time, lower estimated blood loss, lower conversion rate, shorter length of stay, and reduced rates of POPF, bile leak, and delayed gastric emptying. During the early phase, RPD had a longer operative time but a shorter length of stay and a lower CR-POPF than LPD. In the late phase, RPD showed lower conversion, while most other outcomes were comparable.

**Conclusion:**

Both RPD and LPD improve with experience. RPD may offer selected learning-phase advantages, but current evidence does not support broad superiority over LPD.

**Supplementary Information:**

The online version contains supplementary material available at 10.1007/s00423-026-04108-0.

## Introduction

Minimally invasive pancreatoduodenectomy (MIPD), including laparoscopic pancreatoduodenectomy (LPD) and robotic pancreatoduodenectomy (RPD), is increasingly performed in high-volume centers [1–3]. However, it remains one of the most technically demanding minimally invasive procedures because it combines complex retroperitoneal dissection, vascular-adjacent operative steps, lymphadenectomy, and multiple technically precise reconstructions. As a result, safe implementation depends not only on surgical expertise but also on structured training, institutional volume, and progression along the learning curve [4].

International consensus statements have emphasized these requirements. The Miami International Evidence-based Guidelines on Minimally Invasive Pancreas Resection highlighted the need for careful patient selection, adequate center volume, structured implementation, and learning-curve awareness in MIPD, including pancreatoduodenectomy [5]. More recently, the Brescia Internationally Validated European Guidelines on Minimally Invasive Pancreatic Surgery updated these recommendations and specifically addressed laparoscopic and robotic pancreatic surgery as distinct modalities, reflecting the rapid expansion of robotic procedures and the need for procedure-specific guidance [6]. In addition, the 2023 International Consensus Guidelines on Robotic Pancreatic Surgery stated that RPD, in experienced hands, may be associated with lower estimated blood loss and shorter hospital stay compared with open surgery, while also underlining the continued need for comparative studies against laparoscopic approaches and for stronger evidence regarding training and implementation [7].

Despite these advances, the comparative learning-curve behavior of RPD and LPD remains insufficiently defined. LPD is an established minimally invasive approach but is limited by rigid instrumentation, restricted intracorporeal suturing flexibility, and challenging ergonomics. RPD may overcome some of these constraints through wristed instruments, stable three-dimensional visualization, tremor filtration, and improved dexterity, particularly during pancreaticojejunostomy, hepaticojejunostomy, vascular-adjacent dissection, and hemostasis [8, 9]. These theoretical advantages may be especially relevant during the learning phase, yet whether they translate into more favorable operative maturation compared with LPD remains unclear [10].

Existing literature has mainly evaluated the feasibility, safety, and outcomes of either RPD or LPD, often without explicitly separating early and late learning phases. Training studies such as the multicenter LAELAPS-3 program demonstrated that structured RPD training can be implemented safely in selected high-volume centers without an adverse learning-curve impact on clinical outcomes [11, 12]. However, pooled evidence comparing how operative performance and postoperative outcomes evolve across learning phases in RPD versus LPD remains limited. This represents a clinically relevant gap because learning-phase performance may influence program design, credentialing, proctoring, patient selection, and the safe adoption of MIPD.

Therefore, this systematic review and meta-analysis aimed to evaluate modality-specific learning-curve effects in minimally invasive pancreatoduodenectomy. The primary endpoint was operative time, selected as a sensitive marker of technical maturation and team efficiency. Secondary endpoints included estimated blood loss, conversion to open surgery, postoperative length of stay, readmission, overall morbidity, and pancreatoduodenectomy-specific complications. By integrating early-versus-late phase comparisons within each modality and direct RPD-versus-LPD comparisons within early and late learning strata, this study sought to clarify whether the robotic platform confers measurable learning-phase advantages over conventional laparoscopy in minimally invasive pancreatoduodenectomy.

## Methods

### Protocol and reporting guidelines

The review protocol was registered in PROSPERO (CRD420261283406) and conducted in accordance with Cochrane methodology and PRISMA guidelines [13, 14]. Methodological quality was further ensured through adherence to the AMSTAR 2 framework [15].

### Search strategy

**Fig. 1:**
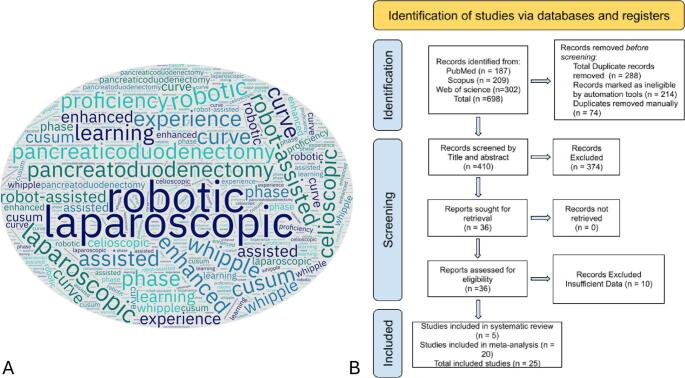
Database search and PRISMA (**A**) Word Cloud (**B**) PRISMA Flowchart

We searched PubMed, Scopus, and Web of Science through June,1 2026, using keywords: (pancreatoduodenectomy OR pancreaticoduodenectomy OR Whipple) AND (robotic enhanced OR robotic OR robot-assisted) AND (laparoscopic OR laparoscopic assisted OR celioscopic) AND (learning curve OR experience OR phase OR proficiency OR cusum), as demonstrated by the word cloud (Fig. 1A).

### Study selection and eligibility criteria

The primary research questions and inclusion criteria were formulated using the PICO framework, targeting a population of patients indicated for PD. The intervention and comparison arms evaluated both the surgical approach (robotic versus laparoscopic) and the stage of the procedural learning curve (early versus late phase). Accordingly, studies were eligible for inclusion if they provided comparative intraoperative or short-term postoperative data for at least one of the following scenarios: early versus late phases within either the robotic or laparoscopic approach, or a head-to-head comparison between RPD and LPD strictly within their respective early or late phases.

To identify eligible literature, two independent investigators (M.M., Z.H.) executed a standard two-stage screening process, initially evaluating titles and abstracts to exclude irrelevant records before conducting a comprehensive full-text review. Non-comparative case series, review articles, case reports, animal or simulator studies, and publications lacking extractable phase-specific data were excluded. Any inter-reviewer discrepancies during the screening process were successfully resolved via consensus or through adjudication by a third reviewer.

### Data extraction

Two independent investigators (M.M., Z.H.) extracted data utilizing a standardized Excel spreadsheet. Any discrepancies were resolved through consensus or adjudication by a third reviewer.

Extracted intraoperative variables included operative time (OT), estimated blood loss (EBL), harvested lymph nodes (HLN), and rates of conversion to open surgery. Short-term postoperative outcomes comprised major complications (defined as Clavien-Dindo grade ≥ III) and postoperative pancreatic fistula (POPF) [16]. In accordance with the 2016 updated consensus definition established by the International Study Group on Pancreatic Surgery (ISGPS), postoperative pancreatic fistula (POPF) data were rigorously stratified to delineate transient biochemical leaks — reclassified as biochemical leak (BL) and excluded from true fistula designation — from clinically relevant fistulas, with only the latter (grades B and C) being extracted for further analysis [17]. Additional extracted postoperative parameters included delayed gastric emptying, post-pancreatectomy hemorrhage, bile leak, surgical site infection (SSI), length of hospital stay (LOS), and rates of reoperation and readmission.

To systematically evaluate surgical evolution and mitigate learning curve bias across the minimally invasive modalities, outcomes for the robotic and laparoscopic arms were extracted and stratified into early and late phases. The number of cases required to overcome the learning curve was extracted as defined by each primary study. Where primary studies did not explicitly define the learning curve threshold, cases were dichotomized based on the standardized phase model proposed by Müller et al. [18]. Accordingly, the early phase (competency) was defined as the initial 39 cases for the laparoscopic arm and 25 cases for the robotic arm. All subsequent cases beyond these procedural thresholds were categorized into the late phase (encompassing proficiency and mastery). Individual data points digitally extracted from graphical plots were converted into sample sizes, means, and standard deviations using the Meta-Analysis Accelerator platform [19].

### Quality assessment

Methodological quality was independently assessed based on study design. Retrospective and prospective observational cohorts were evaluated utilizing the Newcastle-Ottawa Scale (NOS), with a score of ≥ 7 defining a low risk of bias. Randomized controlled trials were evaluated utilizing the Cochrane Risk of Bias 2 (RoB 2) tool across its five standardized domains. Methodological quality was not utilized as a strict exclusion criterion during the initial screening phase; however, studies demonstrating a critically high risk of bias (e.g., NOS score < 4) following full-text evaluation were a priori planned for exclusion from the quantitative synthesis [20, 21].

### Certainty of evidence

Evidence certainty was assessed using the GRADE framework, with baseline ratings set as high for randomized trials and low for observational studies. Two independent reviewers (O.A., Y.B.) evaluated risks of bias, inconsistency, indirectness, imprecision, and publication bias, resolving disagreements by discussion or third-reviewer consultation (K.M.). Final certainty levels are reported as high, moderate, low, or very low [22].

### Statistical analysis

Statistical analyses were performed using R software (version 4.5.2) with the meta package. Continuous outcomes were evaluated using Mean Differences (MD), whereas dichotomous variables were assessed using Odds Ratios (OR). All pooled effect sizes were reported alongside 95% Confidence Intervals (CIs). To account for clinical and methodological diversity across the included studies, a random-effects model utilizing the Restricted Maximum Likelihood (REML) estimator was universally applied [23].

Statistical heterogeneity was evaluated via Cochran’s Q test and quantified using the I^2^ statistic, with thresholds of 25%, 50%, and 75% representing low, moderate, and high heterogeneity, respectively. An I^2^ value of 0% was defined as minimal heterogeneity to account for baseline surgical variations, and a *p* < 0.10 or an I^2^ > 50% indicated significant between-study heterogeneity [14].

The number of cases required to overcome the learning curve was assessed for normality utilizing the Shapiro-Wilk test. Due to non-normal data distributions, inflection points are reported as medians with interquartile ranges (IQR). Comparative analysis between the robotic and laparoscopic modalities was conducted utilizing the non-parametric Wilcoxon rank-sum test; p-values were calculated utilizing a normal approximation with continuity correction due to the presence of tied ranks [24].

Prespecified subgroup and interaction analyses were conducted by surgical approach and learning phase, and robustness was assessed using leave-one-out sensitivity analyses.

## Results

### Study selection and characteristics

The search identified 698 records. Twenty-five studies met inclusion criteria for qualitative synthesis, and twenty were included in quantitative meta-analysis, comprising 4764 patients (early phase = 1235; late phase = 3529). RPD was performed in 2972 patients, whereas LPD was performed in 1792. Study characteristics and baseline data are summarized in Tables 1, 2 and 3; Fig. 1 [10, 11, 25–47].

**Table 1:** Study characteristics

Study ID	Country	Design	Period	Population	Number of surgeons
Chao 2023	Taiwan	Retrospective cohort	December 2014 - March 2021	Patients with periampullary tumors, gastric cancer (pancreatic head invasion), or pancreatic lesions <2 cm eligible for MIS pancreaticoduodenectomy. Exclusions: tumors > 10 cm, vascular/organ invasion, bulky lymphadenopathy, severe pancreatitis history, or prior major upper GI surgery.	Single surgeon, supported by surgeons who were experienced with laparoscopic and open pancreatic surgery.
Chen 2025	China	Retrospective cohort	April 2016- December 2023	Patients with resectable benign or malignant periampullary/pancreatic head lesions (ASA III) without vascular invasion or metastasis.	Multiple surgeons Same surgical team
Kim 2022	South Korea	Retrospective cohort	June 2020	Patients undergoing pure LPD or RPD by high-volume surgeons for benign or malignant indications (progressing with surgeon experience). Those with major vessel or adjacent organ invasion were excluded.	3 surgeons
Zhang 2018	China	Retrospective cohort	December 2013- September 2017	Patients with periampullary tumors requiring pancreaticoduodenectomy (excluding locally advanced malignancies) and fit for pneumoperitoneum. The analysis focused on the learning curve experience, comparing the initial 20 consecutive LPD cases against the initial 20 consecutive RPD cases to assess early-stage surgical implementation.	Multiple surgeons
Tyutyunnik 2022	Multicenter European study; Russia, Netherlands, Italy, Germany	Retrospective cohort	January 2007- March 2020	300 consecutive patients undergoing MIPD for malignant and benign tumors of the head of the pancreas and periampullary area.	3 senior surgeons
Nickel 2023	Germany	Retrospective cohort	2007- September 2020	512 patients were included: 256 patients who underwent MIDP (126 LDP, 130 RDP) matched to 256 who had ODP, considering BMI, ASA fitness grade, tumor size, and need for extended resection (multivisceral and/or vascular).	Multiple surgeons
Wehrle 2024	USA	Retrospective cohort	2010–2020	Patients with pancreatic cancer, aged ≥ 18 years, underwent LPD or RPD and had a minimum follow-up of two years.	Multiple surgeons
Zhang 2019	China	Retrospective cohort	February 2012- July 2016	Consecutive patients who underwent RPD for malignant or benign pathologies had resectable tumors confined to the pancreatic head or periampullary region without vascular invasion, ASA score < 3.	Single surgeon, supported by surgeons who were experienced with laparoscopic and open pancreatic surgery
Zhang 2025	China	Retrospective cohort	between 2015 and 2022	Consecutive patients from nine centers in China who underwent R PD or LPD between 2015 and 2022 were included. A propensity score matchin (PSM) was used to minimize bias.	Multiple surgeons: RPD by seven surgeons from 4 centers, LPD by seven surgeons from five centers.
Gall 2020	England	Retrospective cohort	April 2011-May 2019	Patients with pancreatic pathology/tumor indicated for PD via robotoic or laparoscopic or open in an HBP centre (April 2011-May 2019)	Single surgeon
Wei 2022	China	Randomised control trial	May 2017- April 2020	Patients with surgically resectable, non-metastatic benign or early-stage (T1/T2, less than 3 cm) tumors of the pancreatic head, ampullary, periampullary, common bile duct, or duodenum, graded ASA I–III and fit for surgery.	Single Surgeon
Dai 2024	China	Retrospective cohort	April 2020 - Novomber 2022	Patients with pancreatic pathology/tumor indicated for PD via robotoic (April 2020 - Novomber 2022)	Single surgeon
Wang 2024	China	Retrospective cohort	June 2018-June 2022	Patients undergoing successful laparoscopic pancreaticoduodenectomy for tumors around the pancreas head and ampulla (without distant metastasis).	Not specified
Martin 2024	USA	Retrospective cohort	2012–2021	Patients underwent PD for any indication	Single surgeon assisted by senior surgeon
DeLaura 2024	USA	Retrospective cohort	January 2013-January 2021	Patients underwent PD without further vascular reseaction	Single surgeon
Zwart 2021	the Netherlands	prospective multicenter study	March 2016 -October 2019	Patients undergoing robotic pancreatoduodenectomy with BMI below or equal to 35 Kg/m2 without signs of vascular involvement on a pancreatic CT scan not older than 4 weeks at the time of surgery.	15 surgeons
Shyr 2018	Taiwan	prospective cohort study	September 2011 -July 2014 onward	Patients of robotic pancreatic surgeries, all of the cases were pure robotic pancreatic surgery, with exclusion of cases needing conversion or hybrid surgery.	multiple surgeons
Fu 2026	China	retrospective cohort study	July 2016 - August 2024	302-patient cohort who underwent RPD by a single surgeon (X.Y.Y.)	one single surgeon
Castel 2025	France	Retroscpective cohort	May2018-October2023	Patients indicated to PD due to benign or malignant conditions	Single Surgeon
Napoli 2016	Italy	Retrospective cohort	April 2008-July2014	Patients with pancreatic benign or malignant tumors given that there’s no distant metassis	Single Surgeon
Shi 2019	China	Retrospective cohort	May2010-December2018	patients with resectable pancreatic, ampullary, or bile duct tumors smaller than 10 cm, provided they had no major vascular invasion and were physically fit for anesthesia. Exclusion criteria primarily focused on patients with a history of upper abdominal surgery, tumors exceeding 10 cm, or those involving significant vascular invasion or general anesthesia intolerance	3 surgeons
Marino 2020	Spain	Retrospective cohort	March2014-October2016	patients with small pancreatic head tumors (≤ 6 cm) and no major vascular involvement who were physically fit for anesthesia (ASA score ≤ 3). Key exclusions included patients with metastatic disease, those requiring vascular reconstruction or palliative bypass, and individuals with a BMI exceeding 35 kg/m²	Single surgeon
Xu 2025	china	Retrospective cohort	January 2016 – December 2022	Consecutive adult patients (> 18 years) with pathologically confirmed distal cholangiocarcinoma (DCC) who underwent curative minimally invasive pancreaticoduodenectomy (LPD or RPD) at 8 high-volume Chinese centers. Total cohort: 529 patients (251 LPD, 278 RPD); after 1:1 PSM: 454 patients (227 LPD, 227 RPD)	Multiple surgeons
Emmen 2024	Netherlands	Single-center observational cohort study; post-hoc assessment of a prospectively maintained database	January 2010 – February 2022	All consecutive minimally invasive hepato-pancreato-biliary (MIS-HPB) resections performed at Amsterdam UMC. Among 1,875 pancreatic and liver resections, 600 MIS-HPB resections were included (291 pancreatic resections and 309 liver resections), including laparoscopic and robotic procedures and conversions.	Multiple surgeons
Kang 2024	South Korea	Retrospective cohort	January 2015 – December 2020	Adults with benign or malignant periampullary tumors (pancreatic, ampullary, and distal common bile duct) undergoing pancreatoduodenectomy at two tertiary academic centers (Seoul National University Hospital and Seoul National University Bundang Hospital)	Multiple surgeons

**Table 2:** Patients’ baseline characteristics of Early vs. Late comparison

Study ID	Approach	Number		Sex (F/M)		Age / years (mean ± SD)		BMI / Kg/m2 (mean ± SD)		Comorbitities					
										Cardiovascular disease		DM		Previous abdominal surgery	
		Early	Late	Early	Late	Early	Late	Early	Late	Early	Late	Early	Late	Early	Late
Chao 2023	Robotic	18	57	33/42	NR	67.26 ± 13.28	NRN	24.54 ± 3.57	NR	NR	NR	NR	NR	NR	NR
	Laparoscopic	13	26	13/0	15/11	59.6 ± 11.2	61.8 ± 10.6	23.28 ± 3.32	NR	NR	NR	NR	NR	NR	NR
Zhang 2018		40	60	18/22	27/33	59.6 ± 11.2	61.8 ± 10.6	22.6 ± 3.1	23.1 ± 3.4	6 (15%)	11 (18.33%)	7 (17.5%)	16 (26.67%)	5 (12.5%)	10 (16.67%)
Zhang 2019	Robotic	51	15	19/9	32/6	63.1	68.3	NR	NR	NR	NR	NR	NR	NR	NR
Lim 2020	Robotic	30	30	14/16	17/13	68 ± 14	71 ± 11.5	24 ± 3.4	27.2 ± 5.5	NR	NR	NR	NR	5 (16.7%)	10 (33.3%
Marino 2020	Robotic	33	67	44 / 56	NR	NR	NR	NR	NR	9 (9%)	N	28 (28%)	NR	29 (29%)	NR
Dai 2024	Robotic	111	5	NR	NR	68(61–74)	NR	24.9	NR	NR	NR	NR	NR	NR	NR
Emmen 2022	Robotic	52	123	NR	NR	70(58–78)	66(54–73)	24.2	26.3	NR	NR	NR	NR	NR	NR
	Laparoscopic	33	37	17 / 16	23 / 14	58.9 ± 12.5	61.2 ± 14.2	23.8 ± 3.3	22.9 ± 2.8	1 (3.0%)	2 (5.4%)	3 (9.1%)	3 (8.1%)	21 (63.6%)	17 (51.5%)
Napoli 2016	Robotic	100	350	39/61	162/188	56.8	58.3	23	23.2	NR	NR	NR	NR	0	0
Shi 2019	Robotic	20	41	(11/9)	(18/22)	65 ± 13	65 ± 13	23.6 ± 2.9	24.1 ± 4.0	NR	NR	NR	NR	NR	NR
Shyr 2018	Robotic	35	85	13/22	38/47	581 ± 8.8	60.0 ± 8.9	18.5 ± 2.8	18.4 ± 2.7	4 (11.4%)	13 (15.3%)	2 (5.7%)	6 (7.1%)	5 (14.3%)	10 (11.8%)
Wang 2024	Laparoscopic	16	44	46242	17/27	55.74 ± 3.45	57.89 ± 177	22.99 ± 0.86	22.88 ± 0.42	NR	NR	NR	NR	NR	NR
wei 2022	Robotic	120	182	NR	NR	NR	NR	NR	NR	NR	NR	NR	NR	NR	NR
Fu 2026	Robotic	40	60	18/22	29/31	54.68 ± 11.59	55.3 ± 15.72	23.05 ± 3.09	24.34 ± 3.87	NR	NR	NR	NR	NR	NR
Zhang 2019	Robotic	83	144	39/44	70/74	65.0 ± 12.6	67.0 ± 10.6	26.0 ± 5.0	25.0 ± 3.6	138 (50.2%)		46 (16.7%)	NR	42 (15.3%)	NR
Zwart 2021	Robotic	40	20	15/25	46364	67.6 ± 10.8	62.1 ± 12.5	24.0 ± 3.7	24.2 ± 3.7	NR	NR	NR	NR	23 (57.5%)	13 (65.0%)
Martin 2024	Laparoscopic	90	20	46/43	13/7	67 ± 11.1	66 ± 8.15	25.8 ± 4.6	29.1 ± 2.4	NR	NR	16(17.8%)	0	42 (47.2%)	11 (57.9%)
Castel 2025	Robotic	40	20	15/25	46364	67.6 ± 10.8	62.1 ± 12.5	24.0 ± 3.7	24.2 ± 3.7	N	NR	NR	NR	23 (57.5%)	13 (65.0%)

Study ID	Operation		ASA score				Tumor size/ cm (mean ± SD)		Histopathology			
			I-II		III-IV				Benign		Malignant	
	Whipple	PSPD	Early	Late	Early	Late	Early	Late	Early	Late	Early	Late
Chao 2023	NR	NR	35 (60%)	NR	30 (40%)	NR	2 ± 1.48	NR	23 (30.7%)	NR	52 (69.3%)	NR
	7 (17.9%)	32 (82.1%)	24 (61.5%)	NR	15 (38.5%)	NR	2.81 ± 2.69	NR	12 (30.8%)	NR	27 (69.2%)	NR
Zhang 2018	NR	NR	40(100%)	60 (100%)	0	0	2.4 ± 1.4	2.9 ± 1.6	10 (25%)	12 (20%)	30 (75%)	48 (80%)
Zhang 2019	NR	NR	NR	NR	NR	NR	NR	NR	NR	NR	NR	NR
Lim 2020	NR	NR	27 (90%)	26 (86.7%)	3 (10%)	4 (13.3%)	2.8 ± 1.5	3.1 ± 2.8	NR	NR	NR	NR
Marino 2020	76 (76%)	24 (24%)	82 (82%)	NR	18 (18%)	NR	NR	NR	26 (26%)	NR	74 (74%)	NR
Dai 2024	111	5	75(67.6%)	NR	36(32.4%)	NR	2.3	NR	NR	NR	63(56.8%)	NR
Emmen 2022	52	123	42(80.8%)	90(73.2%)	10(19.2%)	33(26.8%)	1.8	3	NR	NR	33(63.5%)	25(20.3%)
	NR	NR	NR	NR	NR	NR	NR	NR	13 (39.4%)	14 (37.8%)	20 (60.6%)	23 (62.2%)
Napoli 2016	NR	NR	97(97%)	334(95.4%)	3(3%)	16(4.6%)	2.75	2.73	56(56%)	162(43.2%)	44(44.0%)	188(53.7%)
Shi 2019	NR	NR	NR	NR	NR	NR	NR	NR	NR	NR	NR	NR
Shyr 2018	NA	NA	20 (57.1%)	53 (62.3%)	15 (42.9%)	32 (37.7%)	2.4 ± 1.1	2.5 ± 0.9	0	0	35 (100%)	85 (100%)
Wang 2024	NR	NR	13 (I) 3 (II)	29 (I) 15 (II	0	NR	NR	NR	NR	NR	NR	NR
wei 2022	NR	NA	193 (63.91%)	NR	109 (36.09%)	NR	NR	NR	NR	NR	NR	NR
Fu 2026	NR	NR	40 (40%)	60 (60%)	0	0	2.4 ± 1.4	2.9 ± 1.6	12 (17%)	10 (25%)	57 (83%)	30 (75%)
Zhang 2019	NR	NR	62 (74.7%)	103 (71.5%)	21 (25.3%)	42 (29.2%)	2.6 ± 1.3	NR	19 (22.8%)	36 (25.0%)	64 (77.2%)	108 (75.0%)
Zwart 2021	NR	NR	36 (90.0%)	20 (100%)	4 (10.0%)	0	2.2 ± 1.1	2.6 ± 1.7	11 (27.5%)	3 (15.0%)	29 (72.5%)	17 (85.0%)
Martin 2024	NR	NR	NR	NR	NR	NR	NR	NR	NR	NR	59 (65.6%)	9 (45.0%)
Castel 2025	Nr	Nr	36 (90.0%)	20 (100%)	4 (10.0%)	0	2.2 ± 1.1	2.6 ± 1.7	11 (27.5%)	3 (15.0%)	29 (72.5%)	17 (85.0%)

**Table 3:** Patients’ baseline characteristics of Robotic vs. laparoscopic early and lates phase comparisons

Study ID	Approach	Number	Sex (F/M)	Age / years (mean	BMI (kg/m2)	Diabetes, n (%)	Previous abdominal surgery
Chao 2023	Robotic	65	33/42	67.3 ± 13.3	24.5 ± 3.6	NR	NR
	Laparoscopic	39	28/11	66 ± 12.1	23.3 ± 3.3	NR	NR
Chen 2025	Robotic	54	20/34	60.6 ± 9.9	22.5 ± 2.8	13 (24.1%)	3 (5.6%)
	Laparoscopic	36	14/22	56.5 ± 17.9	22.9 ± 3.2	3 (8.3%)	3 (8.3%)
Kim 2022	Robotic	32	15/17	59.7 ± 9.9	23.2 ± 1.9	10 (31.3%)	3 (9.4%)
	Laparoscopic	32	12/20	60.7 ± 12.4	23.3 ± 2.6	7 (21.9%)	4 (12.5%)
Tyutyunnik 2022	Robotic	100	57/43	57.2 ± 12.7	NR	22 (22%)	57 (57%)
	Laparoscopic	100	58/42	59.3 ± 10.32	NR	22 (22%)	22 (22%)
Zhang 2018	Robotic	20	46246	66 ± 7	24.8 ± 2.5	3 (15%)	NR
	Laparoscopic	20	46276	61.5 ± 8.5	24 ± 3.5	4 (20%)	NR
Zhang 2019	Robotic	100	47/53	55.1 ± 14.2	23.8 ± 3.6	NR	NR
Wehrle 2024	Robotic	625	302/323	66.5 ± 10.4	NR	NR	NR
	Laparoscopic	625	293/332	65.6 ± 10.1	NR	NR	NR
Nickel 2023	Robotic	130	NA	59.2 ± 13.7	26.7 ± 4.8	21 (16.2%)	NR
	Laparoscopic	126	NA	60.4 ± 14.8	26.7 ± 4.6	20 (15.9%)	NR
Zhang 2025	Robotic	1006	394 / 612	60.5 (52.0–67.0)	23.4 (21.3–25.2)	NR	NR
	Laparoscopic	1006	384 / 622	61.0 (52.0–67.0)	23.1 (20.9–25.5)	NR	NR
Gall 2020	Robotic	25	46281	60.93 ± 12.52	NR	NR	NR
	Laparoscopic	41	18/23	65.18 ± 11.36	NR	NR	NR
Dai 2024	Robotic	100	44/56	62.5 (53–67.3)	22.54 (20.26–25.06)	28 (28%)	29 (29%)
DeLaura 2024	Robotic	69	38/31	67.04 ± 11.11	28.86 ± 7.44	NR	NR
	Laparoscopic	17	19/7	66.84 ± 8.46	29.76 ± 7.76	NR	NR
Shyr 2018	Robotic	61	(29/32)	65 ± 13	24.0 ± 3.7	NR	NR
Fu 2026	Robotic	302	(132/170)	< 60 173 (57.28), ≥ 60 129 (42.72)	NR	36 (11.92%)	NR
Kang 2024	Robotic	332	147/185	63.6 ± 12.1	23.5 ± 2.6	NR	NR
	Laparoscopic	178	84/94	67.5 ± 11.8	24.3 ± 2.9	NR	NRN
Xu 2025	Robotic	278	104/174	Means not reported; provided categorically:	23.23 ± 2.44	NR	NR
	Laparoscopic	251	93/153	Means not reported; provided categorically:	23.77 ± 3.56	NR	NR

Study ID	Pylorus preservation	Vascular resection	Tumor size (mean + SD)	Histopathology		Pancreatic duct diameter	
				Benign	Malignant	< 3 mm	> = 3 mm
Chao 2023	64 (98.5%)	6 (9%)	2 ± 1.5	23 (35.4%)	52 (80%)	57 (87.7%)	18 (27.7%)
	32 (82.1%)	6 (16.7%)	2.8 ± 2.7	12 (30.8%)	27 (69.2%)	24 (61.5%)	15 (38.5%)
Chen 2025	9 (16.7%)	5 (9.3%)	2.5 ± 1.3	12 (22.2%)	42 (77.8%)	NR	NR
	14 (38.9%)	2 (5.6%)	2.9 ± 1.9	16 (44.4%)	20 (55.6%)	NR	NR
Kim 2022	NR	NR	NR	NR	NR	NR	NR
	NR	NR	NR	NR	NR	NR	NR
Tyutyunnik 2022	NR	4 (4%)	NR	25 (25%)	75 (75%)	NR	NR
	NR	3 (3%)	NR	16 (16%)	84 (84%)	NR	NR
Zhang 2018	NR	NR	NR	NA	NA	11 (55%)	9 (45%)
	NR	NR	NR	NA	NA	7 (35%)	13 (65%)
Zhang 2019	0	0	2.7 ± 1.54	22(22%)	78(78%)	NR	NR
Wehrle 2024	56 (9.0%)	NA	NA	NA	NA	NR	NR
	45 (7.2%)	NA	NA	NA	NA	NR	NR
Nickel 2023	NR	11 (8.5%)	2.9 ± 2.25	64 (49.2%)	66 (50.8%)	NR	NR
	NR	16 (12.7%)	3.11 ± 2.61	62(49.2%)	64 (50.8%)	NR	NR
Zhang 2025	NR	NR	NR	187 (18.6%)	819 (81.4%)	NR	NR
	NR	NR	NR	196 (19.5%)	810 (80.5%)	NR	NR
Gall 2020	NR	NR	23.30 ± 11.16	5	20	NR	NR
	NR	NR	26.56 ± 12.55	6	35	NR	NR
Dai 2024	NR	8 (8%)	NA	26(26%)	74 (74%)	NR	NR
DeLaura 2024	NR	NR	2.59 ± 1.36	7 (10%)	62 (90%0	NR	NR
	NR	NR	3.15 ± 1.84	1 (6%)	16 (94%)	NR	NR
Shyr 2018	NR	NR	NR	NR	NR	NR	NR
Fu 2026	NR	22 (7.28%)	25 (17–34)	130 (43.05%)	172 (56.95%)	3 (2–4)	
Kang 2024	NR	NR	NR	165 (49.7%)	167 (50.3%)	NR	NR
	NR	NR	NR	27 (15.2%)	151 (84.8%)	NR	NR
Xu 2025	NR	8 (2.9%)	2.07 ± 0.89	0 (0%)	278 (100%)	NR	92 (36.7%)
	NR	3 (1.2%)	2.17 ± 1.11	0 (0%)	251 (100%)	NR	104 (37.4%)

### Quality assessment

Following full-text evaluation and rigorous methodological grading, no studies were excluded from the final qualitative or quantitative syntheses based on quality assessment, as all included studies demonstrated a moderate to low risk of bias. Among the 24 observational studies evaluated via the Newcastle-Ottawa Scale, 12 demonstrated a low risk of bias (scores 7–9) [11, 25, 27, 28, 31–33, 37–39, 42, 47]. Moderate risk of bias was observed in 12 studies (score 6) [10, 26, 29, 30, 35, 36, 40, 41, 43–46]. The single randomized controlled trial was evaluated using the Cochrane RoB 2 tool and was judged to have ‘some concerns’ for overall risk of bias, primarily driven by the inherent inability to blind operating surgeons to the surgical platform and the lack of independent, blinded outcome assessors [34].

### Quality of evidence (GRADE assessment)

The certainty of evidence was rated moderate for OT and EBL in the early versus late phase, upgraded due to large effect sizes. Moderate certainty was assigned to late-phase conversion rates (RPD versus LPD). Evidence for early-phase CR-POPF and length of hospital stay was rated low, downgraded for risk of bias, inconsistency, and imprecision.

### Procedural learning curve analysis

Quantitative evaluation of the procedural learning curve, across the 25 studies, revealed that the robotic cohort required a median of 31.5 cases (IQR: 20) to achieve clinical competency, whereas the laparoscopic cohort required a median of 35 cases (IQR: 12.5).(Supplementary Fig. 1) A Wilcoxon rank-sum test indicated that this difference in the case volume necessary to overcome the initial learning phase was not statistically significant (*p* = 0.56) [10, 11, 25–47].

### Learning-phase effects: early versus late pancreatoduodenectomy

#### Operative outcomes

**Fig. 2:**
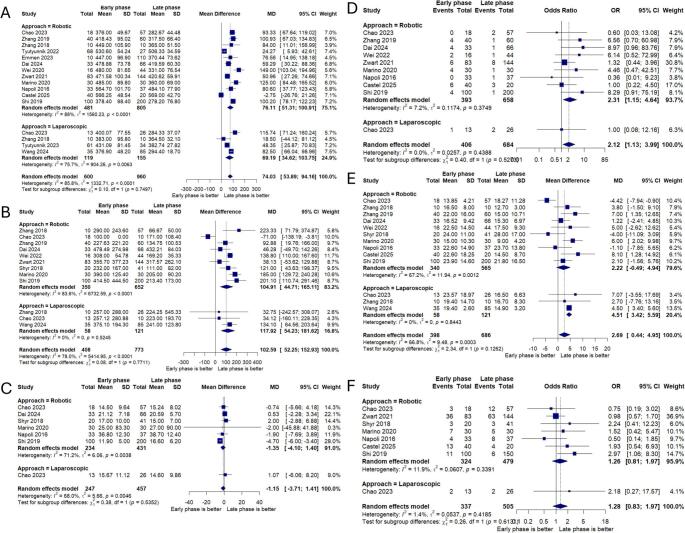
Early versus late phase comparison with robotic and laparoscopic subgroups. (**A**) Operative time (min); (**B**) Estimated blood loss (mL); (**C**) Lymph-node yield; (**D**) Conversion to open surgery; (**E**) Length of hospital stay (days); (**F**) Major complications (CD ≥ III)

Overall, OT was significantly longer in the early phase across 13 studies (*n* = 1,560; early: 600, late: 960) (MD 74.03 min; 95% CI 53.89–94.17; *p* < 0.001; I² = 85.8%; Fig. 2A). This significant reduction in operative time during the late phase remained consistent for both the RPD (MD 71.25 min; 95% CI 46.96–95.54; *p* < 0.001; I² = 90.0%) and LPD cohorts (MD 69.19 min; 95% CI 34.62–103.75; *p* < 0.001; I² = 75.7%) [10, 11, 26, 29–31, 34, 35, 39, 40, 43, 44, 47].

Leave-one-out sensitivity analysis confirmed the robustness of the findings for operative time in both cohorts, with the largest effect size observed after excluding Castel 2025 (MD 83.50 min; 95% CI 61.70–105.30; *p* < 0.0001; I² = 83.1%; Supplementary Fig. 2A) [39]. Similarly, in the LPD group, statistical significance was maintained across all omissions (all *p* < 0.05), with the highest effect size noted after excluding Zhang 2018 (MD 78.53 min; 95% CI 43.60–113.46; *p* < 0.001; I² = 78.7%; Supplementary Fig. 2B) [26].

Estimated blood loss was significantly reduced in the late phase across 10 studies (*n* = 1,181; early: 408, late: 773) (MD 102.59 mL; 95% CI 52.25–152.93; *P* < 0.001; I² = 78.0%), with subgroups confirming this reduction in both RPD and LPD (MD 104.91 mL; 95% CI 44.71–165.11; *p* < 0.001; I² = 83.6.5%; MD 117.92 mL; 95% CI 54.23 to 181.62; *p* < 0.001; I^2^= 0%, respectively)(Fig. 2B) [10, 11, 26, 29, 30, 34, 40, 44, 45, 47]. Leave-one-out sensitivity analysis for EBL in RPD confirmed robustness. The largest effect size was observed after excluding Kang et al. (MD -37.20 mL; I² = 0%; Supplementary Fig. 2C) [42].

Lymph-node yield did not differ between phases across 6 studies (*n* = 704; early: 247, late: 457; *p* = 0.34; Fig. 2C) [10, 30, 40, 43–45]. Leave-one-out sensitivity analysis in RPD demonstrated that the largest effect size was observed after excluding Shi et al. (MD -0.26; I² = 0%; supplementary Fig. 2D) [40]. Conversion to open was significantly higher in the early phase across 9 studies (*n* = 1,090; early: 406, late: 684) (OR 2.12; 95% CI 1.13–3.99; *p* = 0.02; I² = 0.0%; Fig. 2D) [10, 11, 29, 30, 34, 39, 40, 43, 44].

### Postoperative outcomes

Late phase showed a significantly shorter LOS across 11 studies (*n* = 1,084; early: 398, late: 686) (MD 2.69 days; 95% CI 0.44–4.95; *p* = 0.02; I² = 66.8%), driven by the LPD subgroup (MD 4.51 days; *P* < 0.001), whereas RPD showed no difference (*p* = 0.11; Fig. 2E) [10, 26, 29, 34, 39, 40, 43–45, 47]. Leave-one-out sensitivity analysis supported robustness. The largest effect size was observed after excluding Chao 2023 (MD 3.20 days; I² = 37.9%; Supplementary Fig. 2E) [30].

**Fig. 3:**
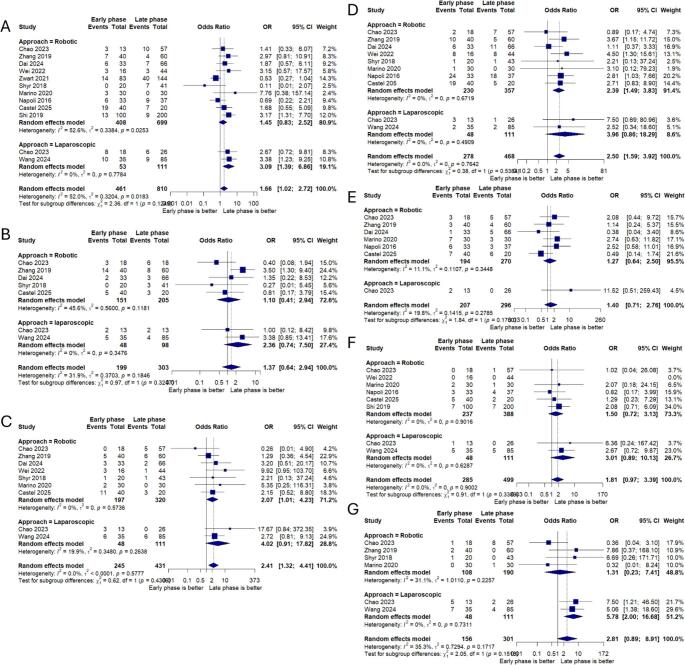
Early versus late phase postoperative complications with robotic and laparoscopic subgroups. (**A**) CR-POPF; (**B**) Post-pancreatectomy hemorrhage (PPH); (**C**) Bile leak; (**D**) Delayed gastric emptying (DGE); (**E**) Readmission; (**F**) Reoperation; (**G**) Surgical site infection (SSI)

Major complication rates were comparable between phases across 7 studies (*n* = 842; early: 337, late: 505; *p* = 0.26; Fig. 2F) [11, 30, 39, 40, 43–45]. However, POPF incidence was significantly elevated in the early phase across 11 studies (*n* = 1,271; early: 461, late: 810) (OR 1.66; 95% CI 1.02–2.72; *p* = 0.043; I² = 52.0%; Fig. 3A) [10, 11, 29, 30, 34, 39, 40, 43–45, 47]. Subgroup analysis demonstrated that this effect was driven by the LPD cohort (OR 3.09; 95% CI 1.39–6.86; I^2^= 0%), while RPD showed no statistically significant difference (OR 1.45; 95% CI 0.83–2.52; *p* = 0.19; I^2^= 52.6%). No significant differences were observed between phases for PPH (6 studies, *n* = 502; early: 199, late: 303; *p* = 0.18; Fig. 3B) [10, 29, 30, 39, 45, 47]. Bile leak was significantly higher in the early phase across 8 studies (*n* = 676; early: 245, late: 431) (OR 2.41; 95% CI 1.32–4.41; *p* = 0.004; I² = 0.0%; Fig. 3C) [10, 29, 30, 34, 39, 44, 45, 47]. DGE was also significantly elevated in the early phase across 9 studies (*n* = 746; early: 278, late: 468) (OR 2.50; 95% CI 1.59–3.92; *p* < 0.001; I² = 0.0%; Fig. 3D) [10, 29, 30, 34, 39, 43–45, 47]. Readmission (6 studies, *n* = 503; early: 207, late: 296; *p* = 0.33; Fig. 3E), reoperation (7 studies, *n* = 784; early: 285, late: 499; *p* = 0.06; Fig. 3F), and SSI (5 studies, *n* = 457; early: 156, late: 301; *p* = 0.08; Fig. 3G) showed no significant differences between early and late phases [10, 29, 30, 34, 39, 43–45, 47].

### Early-phase comparison: RPD versus LPD

#### Operative outcomes

**Fig. 4:**
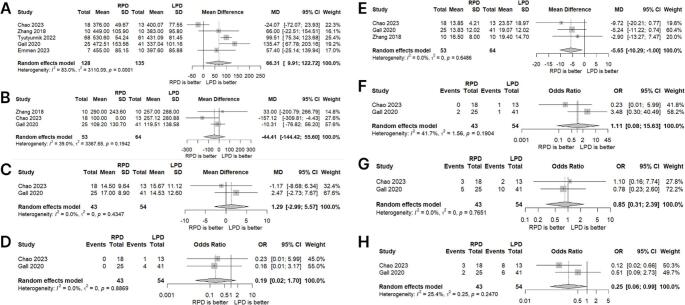
Early learning phase: RPD versus LPD. (**A**) Operative time (min); (**B**) Estimated blood loss (mL); (**C**) Lymph-node yield; (**D**) Conversion to open surgery; (**E**) Length of hospital stay (days); (**F**) Reoperation; (**G**) Major complications (CD ≥ III); (**H**) CR-POPF

During the early learning phase, OT was significantly longer in the robotic cohort across 5 studies comprising 263 patients (RPD = 128; LPD = 135) (MD 66.31 min; 95% CI 9.91-122.72; *p* = 0.02; Fig. 4A) [26, 30, 31, 35, 41]. Considerable between-study heterogeneity was observed (I² = 83.0%), which was eliminated following the exclusion of Chao et al. during sensitivity analysis (I² = 0%; Supplementary Fig. 3A) [30]. Estimated blood loss, evaluated in 3 studies (*n* = 117; RPD = 53; LPD = 64; Fig. 4B), showed no significant difference between the approaches (MD -44.41 mL; 95% CI -144.42 to 55.60; *p* = 0.38), with moderate heterogeneity (I² = 39.0%) [26, 30, 41]. Heterogeneity was eliminated by omitting Chao et al., (Supplementary Fig. 3B) [30]. Harvested lymph nodes were comparable across 2 studies involving 97 patients (RPD = 43; LPD = 54) (MD 1.29; 95% CI -2.99 to 5.57; *p* = 0.55; I² = 0%; Fig. 4C). Similarly, the rate of conversion to open surgery, assessed across 2 studies (*n* = 97; RPD = 43; LPD = 54; Fig. 4D), did not significantly differ between modalities (OR 0.19; 95% CI 0.02 to 1.70; *p* = 0.14; I² = 0%) [30, 41].

#### Postoperative recovery outcomes

Length of hospital stay, analyzed across 3 studies comprising 117 patients (RPD = 53; LPD = 64), was significantly shorter for patients undergoing RPD during the early learning phase (MD -5.65 days; 95% CI -10.29 to -1.00; *p* = 0.017; Fig. 4E), with minimal heterogeneity (I² = 0%) [26, 30, 41]. Reoperation rates, reported in 2 studies involving 97 patients (RPD = 43; LPD = 54), demonstrated no significant difference between the robotic and laparoscopic cohorts, with moderate heterogeneity (OR 1.11; 95% CI 0.08 to15.63; *p* = 0.94; I² = 41.7%; Fig. 4F) [30, 41].

#### Postoperative complications

Rates of major complications (CD ≥ III) were assessed in 2 studies involving 97 patients (RPD = 43; LPD = 54) and were similar between the early learning phases of RPD and LPD (OR 0.85; 95% CI 0.31 to 2.39; *p* = 0.76; I² = 0%; Fig. 4G) [30, 41]. Conversely, the early phase of RPD was associated with a statistically significant reduction in CR-POPF rates across 2 studies (*n* = 97; RPD = 43; LPD = 54) (OR 0.25; 95% CI 0.06 to 0.99; *p* = 0.048; Fig. 4H), demonstrating low between-study heterogeneity (I² = 25.4%) [30, 41].

### Late RPD versus late LPD

#### Operative outcomes

**Fig. 5:**
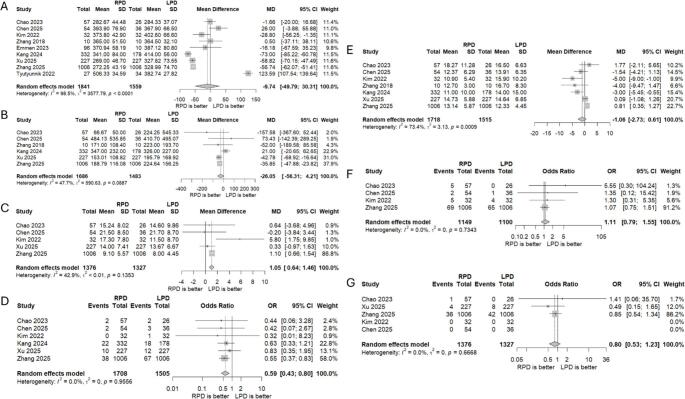
Late learning phase: RPD versus LPD. (**A**) Operative time (min); (**B**) Estimated blood loss (mL); (**C**) Lymph-node yield; (**D**) Conversion to open surgery; (**E**) Length of hospital stay (days); (**F**) Readmission; (**G**) Reoperation

Operative time, analyzed across 9 studies comprising 3,400 patients (RPD = 1,841; LPD = 1,559), demonstrated no significant difference between RPD and LPD during the late learning phase (MD -9.74 min; 95% CI -49.79 to 30.31; *p* = 0.63; Fig. 5A), with considerable heterogeneity (I² = 98.5%). Sensitivity analysis revealed that omitting Tyutyunnik et al. reduced heterogeneity (I² = 91.6%) and yielded a statistically significant reduction in OT favoring RPD (MD -28.71 min; *p* = 0.02; Supplementary Fig. 4A) [25–27, 30, 31, 33, 35, 38, 42]. Estimated blood loss, evaluated in 6 studies involving 3,169 patients (RPD = 1,686; LPD = 1,483), was comparable between the modalities (MD -26.05 mL; 95% CI -56.31 to 4.21; *p* = 0.09; Fig. 5B) with moderate heterogeneity (I² = 47.7%) [25, 27, 30, 33, 38, 42]. However, excluding Kang et al. during sensitivity analysis eliminated heterogeneity (I² = 0%) and demonstrated significantly less EBL in the RPD cohort (MD -37.20 mL; *p* < 0.001; Supplementary Fig. 4B) [42]. Furthermore, late-phase RPD was associated with a significantly higher lymph node yield across 5 studies (*n* = 2,703; RPD = 1,376; LPD = 1,327) (MD 1.05; 95% CI 0.64 to 1.46; *p* < 0.001) with moderate heterogeneity (I² = 42.9%; Fig. 5C) [25, 27, 30, 33, 38]. However, leave-one-out analysis showed that omitting Xu 2025 rendered this difference non-significant (*p* = 0.11; I²=47.3%; Supplementary Fig. 4C) [38]. Additionally, late-phase RPD demonstrated a significantly lower rate of conversion to open surgery across 6 studies (*n* = 3,213; RPD = 1,708; LPD = 1,505) (OR 0.59; 95% CI 0.43–0.80; *p* < 0.001) with minimal heterogeneity (I²=0%; Fig. 5D) [25, 27, 30, 33, 38, 42].

#### Postoperative recovery outcomes

Length of hospital stay was evaluated in 7 studies comprising 3,233 patients (RPD = 1,718; LPD = 1,515) and did not significantly differ between the late-phase cohorts (MD -1.06 days; 95% CI -2.73 to 0.61; *p* = 0.21; Fig. 5E), though substantial heterogeneity was present (I² = 73.4%) [25–27, 30, 33, 38, 42]. Conversely, leave-one-out analysis revealed a significant difference and minimal heterogeneity (MD -37.20; 95% CI -48.06 to -26.33; *p* < 0.001; I^2^= 0; supplementary Fig. 4D) by omitting Kang et al. [42]. Readmission rates, reported across 4 studies involving 2,249 patients (RPD = 1,149; LPD = 1,100), were comparable (OR 1.11; 95% CI 0.79 to1.55; *p* = 0.56; I² = 0%; Fig. 5F). Likewise, reoperation rates assessed in 5 studies comprising 2,703 patients (RPD = 1,376; LPD = 1,327) demonstrated no significant difference between modalities (OR 0.80; 95% CI 0.53 to1.23; *p*5 = 0.31; I² = 0%; Fig. 5G) [25, 27, 30, 33, 38].

#### Post-operative complications

Major postoperative morbidity demonstrated equivalence between the approaches during the proficiency and mastery phases. Specifically, major complications (CD ≥ III) assessed across 6 studies involving 3,213 patients (RPD = 1,708; LPD = 1,505) showed no significant difference (OR 0.98; 95% CI 0.81 to 1.18; *p* = 0.84; I² = 0%; Supplementary Fig. 5A). Similarly, CR-POPF rates were comparable across 5 studies (*n* = 3,213; RPD = 1,708; LPD = 1,505) (OR 0.83; 95% CI 0.67 to 1.04; *p* = 0.10; I² = 0%; Supplementary Fig. 5B). No significant between-group differences were observed for PPH across 5 studies (*n* = 2,651; RPD = 1,337; LPD = 1,314) (OR 0.99; 95% CI 0.75 to 1.32; *p* = 0.97; I² = 0%; Supplementary Fig. 5C), DGE across 5 studies (*n* = 2,703; RPD = 1,376; LPD = 1,327) (OR 0.92; 95% CI 0.73 to 1.16; *p* = 0.49; I² = 0%; Supplementary Fig. 5D), biochemical leak across 5 studies (*n* = 2,703; RPD = 1,376; LPD = 1,327) (OR 0.96; 95% CI 0.72 to1.27; *p* = 0.76; I² = 0%; Supplementary Fig. 5E), or surgical site infections across 2 studies (*n* = 147; RPD = 89; LPD = 58) (OR 2.15; 95% CI 0.50 to 9.18; *P* = 0.30; I² = 0%; Supplementary Fig. 5F) [25, 27, 30, 33, 38, 42].

#### Narrative synthesis of additional studies

Five studies were excluded from quantitative synthesis due to lack of learning-phase–stratified outcomes. DeLaura et al. (2024) reported that prior LPD experience shortened the RPD learning curve for a single surgeon (proficiency at 40 cases), with superior operative time, EBL, conversion, LOS, SSI, DGE, and readmission versus LPD [36]. Fu et al. (2026) analyzed 302 RPD cases by one surgeon, achieving textbook outcome (TO) in 57.0% overall and 63.2% after the learning curve (120 cases); post-learning curve status, diabetes, dilated pancreatic duct (> 3 mm), and ASA ≤ II were independently associated with TO [37]. Wehrle et al. (2024) showed comparable outcomes between RPD and LPD with lower conversion for RPD [28]. Nickel et al. (2023) reported earlier EBL stabilization with robotics in distal pancreatectomy, while Martin et al. (2024) showed abbreviated learning curves for LPD transfer (5 vs. 25 cases) [32].

## Discussion

This systematic review and meta-analysis evaluated modality-specific learning-curve effects in minimally invasive pancreatoduodenectomy (MIPD), focusing on robotic pancreatoduodenectomy (RPD) and laparoscopic pancreatoduodenectomy (LPD). The objective was to determine whether progression from early to late experience improves operative efficiency and perioperative outcomes within each platform, and whether RPD and LPD differ when compared within equivalent learning phases.

The main finding was that progression from the early to the late learning phase was associated with significant improvements in operative performance across MIPD. Operative time and estimated blood loss decreased significantly in the late phase, and these improvements were observed in both RPD and LPD subgroups. Conversion to open surgery was also significantly higher in the early phase, supporting the concept that technical maturation in MIPD is reflected primarily by intraoperative efficiency and procedural completion. These findings are consistent with prior learning-curve studies showing that operative time and blood loss are among the earliest and most sensitive markers of proficiency acquisition in pancreatoduodenectomy. Zhang et al. reported a defined learning curve for robot-assisted laparoscopic pancreatoduodenectomy in a high-volume center, with improvement in operative parameters after the early phase [29]. Tyutyunnik et al. similarly analyzed learning curves across three European centers performing laparoscopic, hybrid laparoscopic, and robotic pancreatoduodenectomy and showed that performance varied substantially across centers and approaches [31]. Dai et al. further demonstrated that even surgeons with extensive LPD experience undergo a measurable RPD learning curve, although previous laparoscopic experience may facilitate robotic adoption [10].

The observed improvement in both RPD and LPD is important because it indicates that technical maturation is not exclusive to the robotic platform. While robotic surgery may offer wristed instrumentation, three-dimensional visualization, tremor filtration, and improved ergonomics, these features should not be interpreted as automatically producing superior outcomes during comparable learning phases [48]. In our analysis, both platforms showed significant early-to-late reductions in operative time and blood loss. This finding supports a more balanced interpretation: structured experience, case accumulation, team standardization, and perioperative pathway maturation appear to be central determinants of improvement in both RPD and LPD.

Postoperative outcomes showed a more selective learning-phase effect. Length of hospital stay was significantly shorter in the late phase overall, but this effect was driven mainly by the LPD subgroup, whereas RPD showed no significant early-versus-late difference. POPF was significantly more frequent in the early phase overall, again driven by the LPD cohort, while no significant difference was observed for RPD. Bile leak and delayed gastric emptying were also significantly higher in the early phase. In contrast, major complications, postpancreatectomy hemorrhage, readmission, reoperation, and surgical site infection did not differ significantly between early and late phases. These findings suggest that some PD-specific complications may improve with increasing experience, particularly those related to reconstruction and postoperative functional recovery, but that global morbidity is influenced by factors beyond the technical platform alone. Pancreatic texture, duct size, patient frailty, vascular involvement, institutional volume, drain management, and ERAS implementation may dilute the measurable effect of the learning phase on postoperative morbidity.

The early-phase comparison between RPD and LPD provides clinically relevant insight into platform-specific implementation. During the early learning phase, RPD was associated with significantly longer operative time than LPD, indicating that robotic setup, docking, console workflow, and robotic reconstruction may initially prolong procedures. Zhang et al. reported longer operative time for robotic-assisted laparoscopic pancreatoduodenectomy compared with total laparoscopic pancreatoduodenectomy, supporting the notion that early robotic implementation may carry an operative time penalty [26]. Kim et al., in a multicenter comparison using propensity score and learning curve-matching analyses, also emphasized the importance of comparing platforms at comparable experience levels rather than assuming equivalence across different adoption stages [25]. In the present study, however, early RPD was associated with shorter length of stay and lower clinically relevant POPF compared with early LPD. This finding may reflect improved precision during pancreatic reconstruction or differences in perioperative pathways at robotic centers, but it should be interpreted cautiously because the certainty of evidence for early-phase CR-POPF and LOS was low and the number of included studies was limited.

In the late phase, RPD and LPD showed broadly comparable outcomes. Operative time and estimated blood loss did not differ significantly in the primary analyses, although sensitivity analyses suggested potential advantages for RPD after excluding individual influential studies. Late-phase RPD was associated with a significantly lower conversion rate compared with late-phase LPD, a finding with moderate certainty and low heterogeneity. This result is consistent with Wehrle et al., who reported comparable oncologic and surgical outcomes between RPD and LPD in pancreatic cancer but observed lower conversion after RPD [28]. Emmen et al., in a pan-European multicenter propensity-matched study, also found broadly comparable outcomes between robot-assisted and laparoscopic pancreatoduodenectomy while identifying differences in specific endpoints rather than global superiority of one platform [35]. In our analysis, late-phase RPD was also associated with higher lymph-node yield, but this association became non-significant after leave-one-out sensitivity analysis, indicating that the finding may be study-dependent rather than robust. Therefore, the late-phase data should be interpreted as showing a clearer advantage for RPD in conversion reduction, but not definitive superiority in operative time, blood loss, morbidity, or postoperative recovery.

The LAELAPS-3 study is particularly relevant to the interpretation of these findings. In this prospective Dutch multicenter training program, 275 RPD procedures were performed across seven centers by 15 trained surgeons under a structured implementation framework including simulation, biotissue drills, video training, and on-site proctorin [11]. Zwart et al. reported that benchmark outcomes could be achieved during implementation and that the learning curve did not negatively affect major clinical outcomes [11]. This supports the concept that RPD can be introduced safely when embedded in structured training and sufficient institutional volume, but it does not imply that the robotic platform alone eliminates the learning curve. Rather, it suggests that training architecture, mentorship, and center-level readiness are critical modifiers of early outcomes.

Several additional learning-curve studies reinforce this interpretation. Chao et al. examined the simultaneous development of laparoscopic and robotic pancreatoduodenectomy and demonstrated that both approaches can be developed in parallel under appropriate institutional conditions [30]. Wang et al. showed that even a proficient laparoscopic surgeon requires a measurable learning period for LPD, emphasizing that general laparoscopic expertise does not fully substitute for procedure-specific PD experience [47]. Jones et al. analyzed international multicenter learning curve-stratified outcomes after RPD and showed that outcomes vary across learning phases, supporting the need to stratify robotic results by experience rather than pooling all cases as a single homogeneous group [49]. Chen et al. used propensity score matching and learning-curve analysis to compare robotic and laparoscopic pancreatoduodenectomy and similarly highlighted that platform comparisons are sensitive to learning-curve stage and institutional experience [27]. Preukschas et al. also emphasized that robotic pancreatic surgery requires structured training and learning-curve assessment rather than unregulated implementation [12].

### Clinical implications

The present findings have practical implications for MIPD implementation. First, both RPD and LPD require structured learning pathways, and neither approach should be introduced without adequate case selection, mentoring, and institutional readiness. Second, early RPD may require longer operative time, which should be anticipated during program planning and operating room scheduling. Third, the lower late-phase conversion rate observed with RPD may represent a clinically meaningful platform-specific advantage in experienced settings, but this advantage should be weighed against costs, access, training burden, and institutional volume. Fourth, because most postoperative complications were comparable between RPD and LPD within the same learning strata, platform selection should not be based solely on theoretical robotic advantages but on local expertise, training infrastructure, and measurable outcome monitoring.

### Strengths

This meta-analysis specifically examined learning-phase effects in minimally invasive pancreatoduodenectomy and separated robotic from laparoscopic approaches. By analyzing early-versus-late outcomes within each modality and comparing RPD versus LPD within early and late strata, the study avoids treating MIPD as a single homogeneous intervention. The inclusion of operative, postoperative, and PD-specific outcomes provides a clinically relevant assessment of both technical maturation and perioperative safety.

### Limitations

Several limitations should be acknowledged. First, most included studies were retrospective cohort analyses, which are inherently susceptible to selection bias, unmeasured confounding, and center-level effects. Second, learning-phase definitions differed across studies, limiting comparability and potentially attenuating or exaggerating pooled estimates. Third, prior laparoscopic, robotic, open pancreatic, and institutional team experience were inconsistently reported, although these factors are likely to influence learning trajectories. Fourth, some subgroup analyses included a limited number of studies and patients, particularly early-phase direct RPD versus LPD comparisons, leading to imprecision and low certainty for selected endpoints such as CR-POPF and LOS. Fifth, substantial heterogeneity was observed in several operative outcomes, particularly operative time, reflecting differences in case complexity, reconstruction techniques, operative workflow, and institutional pathways.Müller et al. emphasized the need for standardized reporting of pancreatic surgery learning curves, as inconsistent definitions limit comparability across studies [18]. Sixth, sensitivity analyses showed that some findings, including late-phase lymph-node yield and selected OT or EBL results, were influenced by individual studies. Finally, long-term oncologic outcomes, quality of life, cost-effectiveness, and time to adjuvant therapy were not uniformly reported and could not be robustly assessed.

## Conclusion

This meta-analysis demonstrates that increasing experience in MIPD is associated with significant improvements in operative time, estimated blood loss, conversion, length of stay, POPF, bile leak, and delayed gastric emptying, while major complications, were largely comparable across learning phases. Early-phase RPD was associated with longer operative time but shorter length of stay and lower CR-POPF compared with early-phase LPD. In the late phase, RPD showed a significantly lower conversion rate and a possible, but non-robust, increase in lymph-node yield, while most other outcomes were comparable between platforms. These findings suggest that robotic pancreatoduodenectomy may provide selected advantages during implementation and proficiency, but they do not support broad superiority over laparoscopy across comparable learning phases. Future prospective multicenter studies should apply standardized learning-curve definitions, account for surgeon- and center-level experience, and evaluate clinically meaningful endpoints beyond operative efficiency to guide training, credentialing, and platform selection in minimally invasive pancreatoduodenectomy.

## Supplementary Information

Below is the link to the electronic supplementary material.

Supplementary Material 1

## Data Availability

All data analyzed during this study are included in this published article and its supplementary information files.

## Abbreviations

PD: Pancreatoduodenectomy
LPD: Laparoscopic pancreatoduodenectomy
RPD: Robotic pancreatoduodenectomy
MIS: Minimally invasive surgery
CR: POPF—Clinically relevant postoperative pancreatic fistula
MD: Mean difference
OR: Odds ratio
CI: Confidence interval
OT: Operative time
EBL: Estimated blood loss
LOS: Length of hospital stay
DGE: Delayed gastric emptying
PPH: Post-pancreatectomy hemorrhage
