# Supplementary material for: Robotic versus laparoscopic pancreatoduodenectomy across the learning curve: a systematic review and meta-analysis
Source: Langenbecks Arch Surg. 2026 Jun 19;411(1):171. doi: 10.1007/s00423-026-04108-0 (PMC13287129; doi:10.1007/s00423-026-04108-0)
Supplement: Supplementary file 1 — Supplementary Material 1 [file 423_2026_4108_MOESM1_ESM.docx]

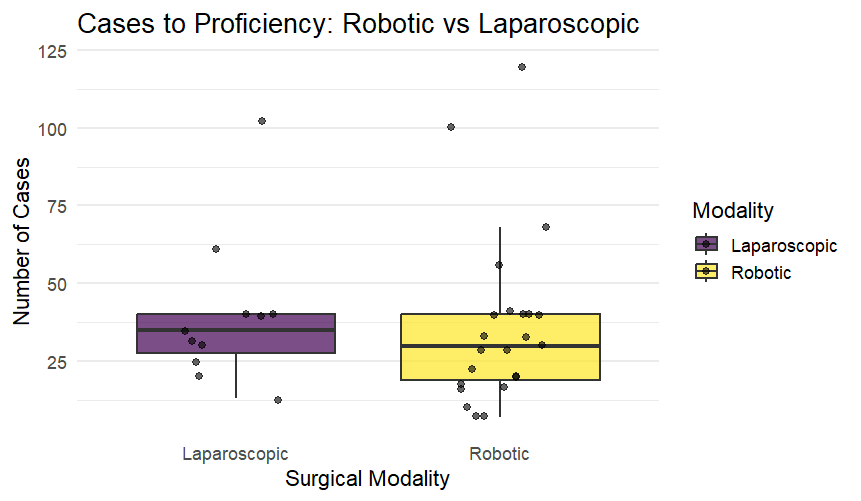


**Supplementary figure1.** A box-and-jitter plot comparing the distribution of the number of cases required to achieve surgical proficiency between laparoscopic and robotic modalities.


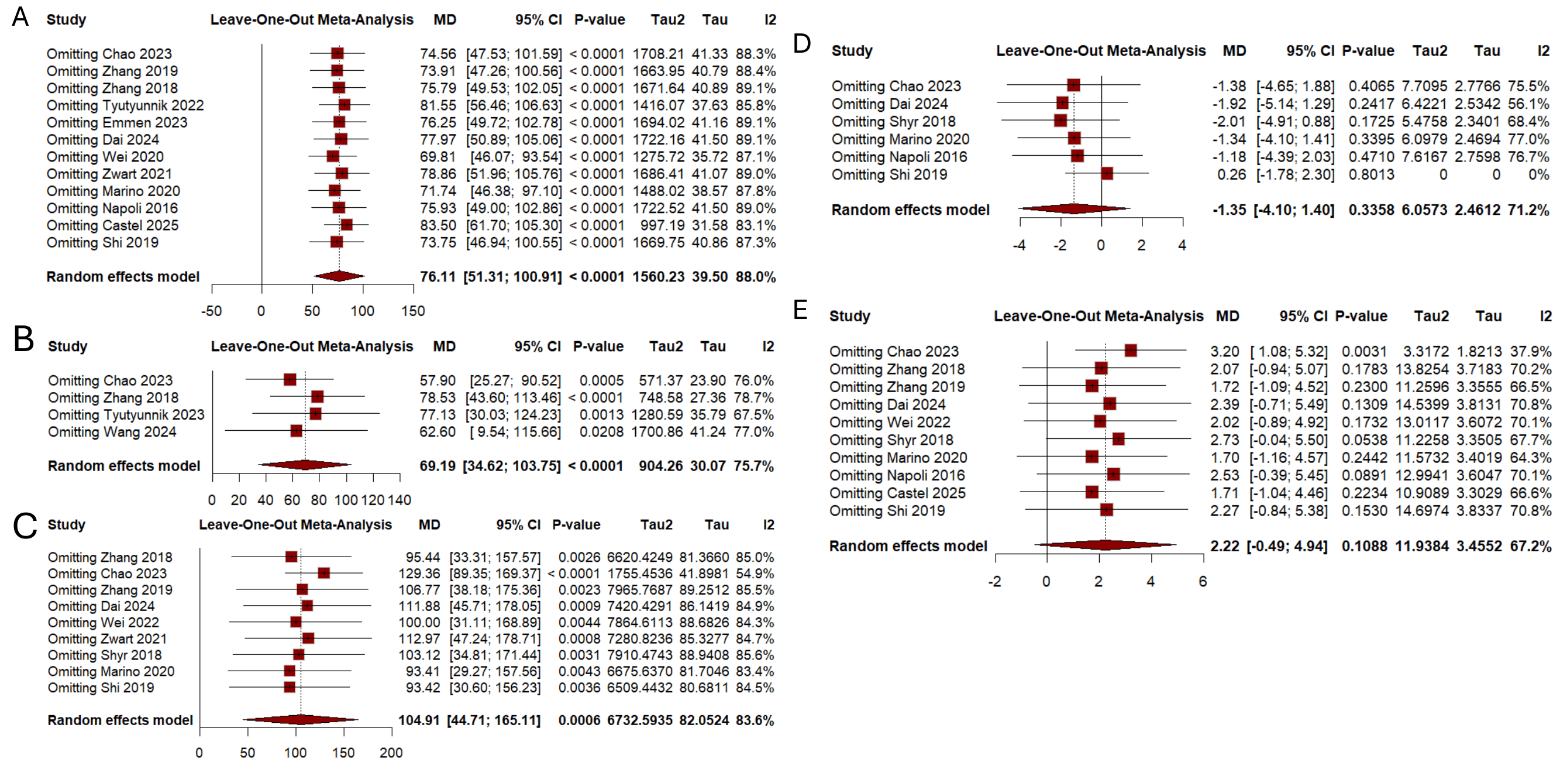


**Supplementary Figure 2.** Leave-one-out sensitivity analysis: Early versus late phase. (A) Operative time — RPD cohort; (B) Operative time — LPD cohort; (C) Estimated blood loss — RPD cohort; (D) Lymph-node yield — RPD cohort (excluding Shi et al.) ;(E) Length of hospital stay—RPD cohort


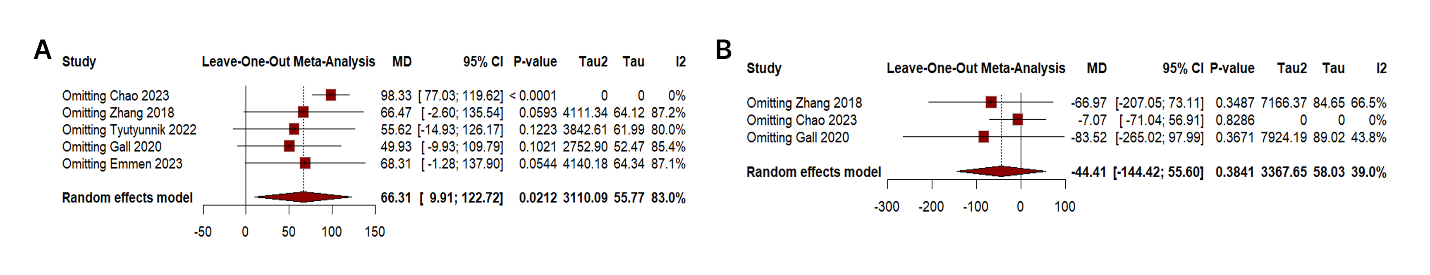


**Supplementary Figure 3.** Leave-one-out sensitivity analysis: Early learning phase RPD versus LPD. (A) Operative time; (B) Estimated blood loss.


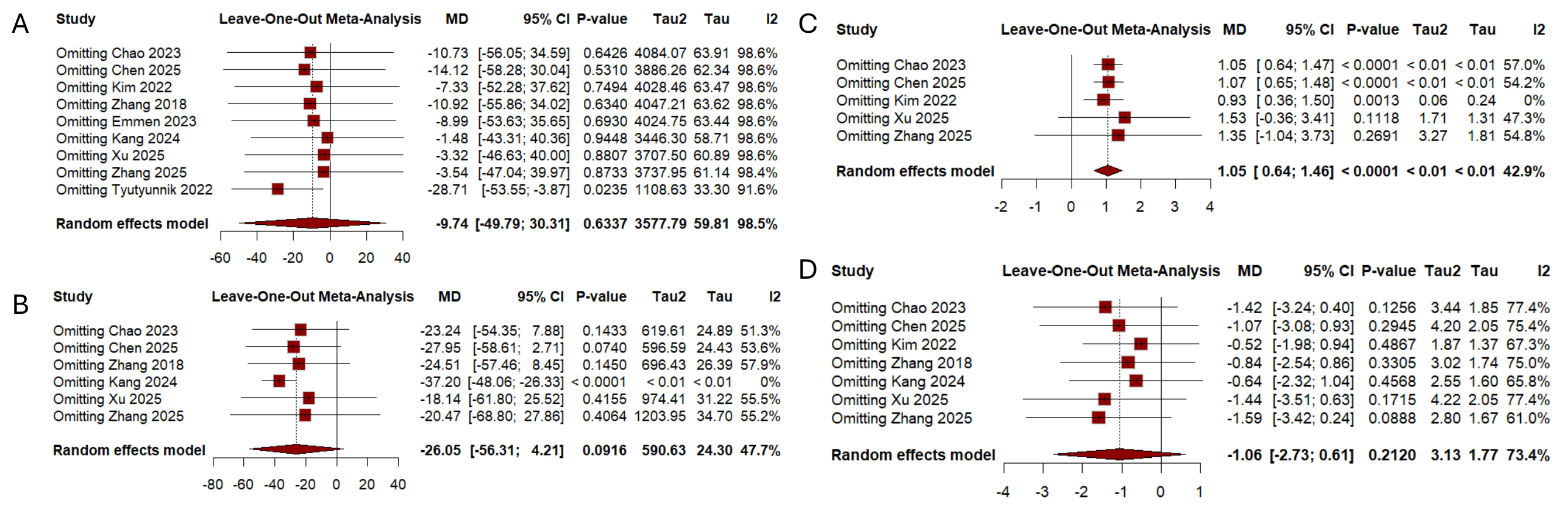


**Supplementary Figure 4.** Leave-one-out sensitivity analysis: Late learning phase RPD versus LPD. (A) Operative time; (B) Estimated blood loss (excluding Kang et al.); (C) Lymph-node yield; (D) Length of hospital stay.


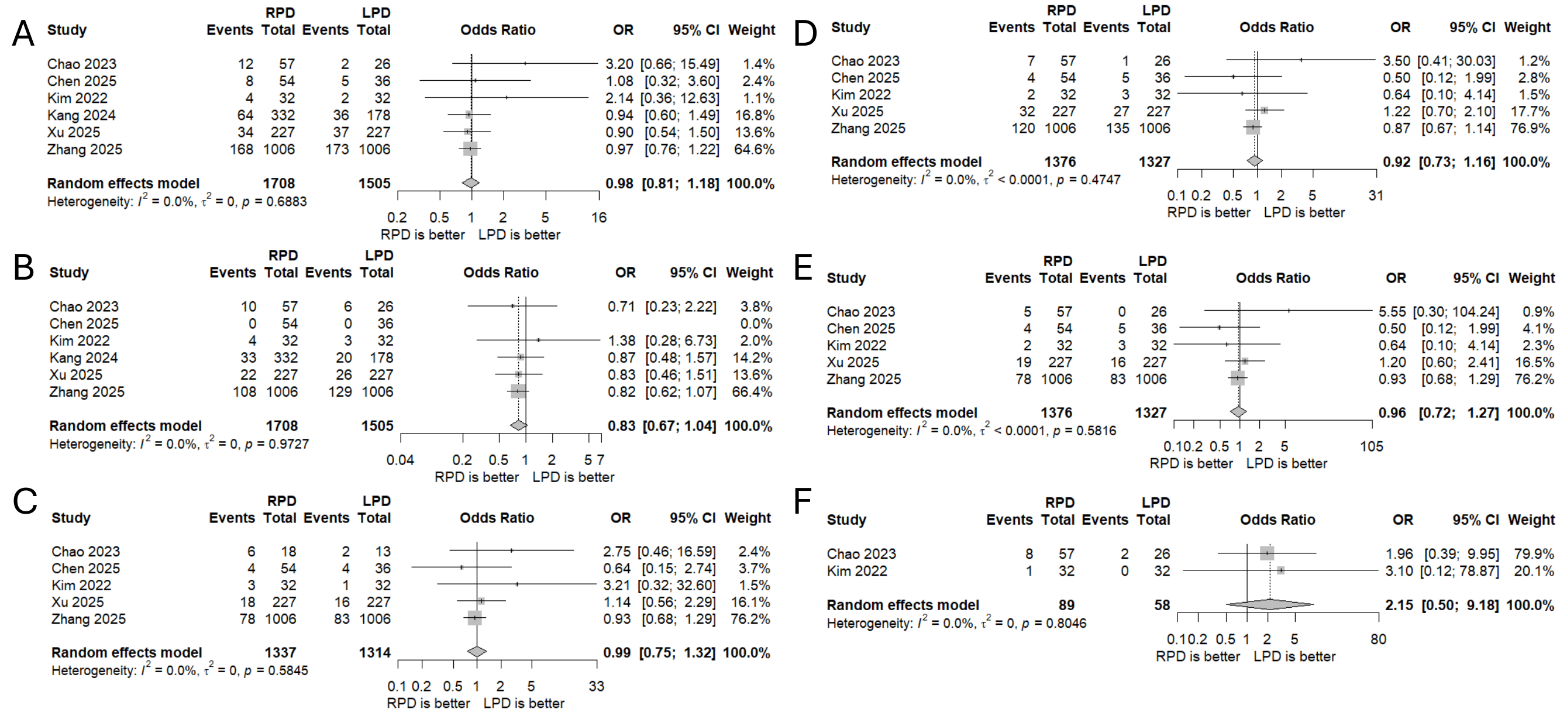


**Supplementary Figure 5**. Late learning phase postoperative complications: RPD versus LPD. (A) Major complications (CD ≥ III); (B) Clinically relevant postoperative pancreatic fistula (CR-POPF); (C) Post-pancreatectomy hemorrhage (PPH); (D) Delayed gastric emptying (DGE); (E) Biochemical leak; (F) Surgical site infection (SSI).
